# Effects of Adjuvant Respiratory Therapy on Secretion Expectoration and Treatment Adherence in Patients with Head and Neck Cancer Receiving Concurrent Chemo-Radiotherapy

**DOI:** 10.3390/medicina61071266

**Published:** 2025-07-13

**Authors:** Hsiu-Ying Cho, Lan-Ti Chou, Chien-Yu Lin, Hsiu-Feng Hsiao, Chun Yu Lin, Horng-Chyuan Lin

**Affiliations:** 1 Department of Respiratory Therapy, Chang Gung Memorial Hospital at Linkou, Taoyuan 33378, Taiwan; 2 Department of Respiratory Therapy, Chang Gung University, Taoyuan 333323, Taiwan; 3 Department of Radiation Oncology, Chang Gung Memorial Hospital at Linkou, Taoyuan 33378, Taiwan; 4 Department of Thoracic Medicine, Chang Gung Memorial Hospital at Linkou, Fu-Hsing Street, Kweishan, Taoyuan 33305, Taiwan; 5 College of Medicine, Chang Gung University, Taoyuan 333323, Taiwan

**Keywords:** adjuvant respiratory therapy, head and neck cancer, concurrent chemo-radiotherapy, secretion expectoration, treatment adherence

## Abstract

*Background and Objectives:* The common complaints of head and neck cancer patients receiving concurrent chemo-radiotherapy (CCRT) are dry mouth, dysphagia, trismus, hoarseness, sore throat, and oral mucosal damage, which result in retained secretions and difficult expectoration. We aimed to investigate the effect of adjuvant respiratory therapy on secretion expectoration and treatment completion in patients with head and neck cancer receiving CCRT. *Materials and Methods:* From November 2016 to May 2018, 56 head and neck cancer patients were recruited retrospectively, and according to their respiratory therapy in the medical record, were divided into the control group (CG, *n* = 27) or the research group (RG, *n* = 29). In the CG, the patients were treated via the teaching of routine breathing exercises and expel techniques, while patients in the RG were treated with the inhalation of a ß-agonist bronchodilator agent five times each week, in addition to the standard treatment administered in the CG. *Results:* The total completion rate of treatment was significantly higher in the RG (21 patients) compared with the CG (12 patients) (72.4% vs. 44.4%, *p* < 0.05). After therapy, the rates of clinical symptoms were significantly increased in the RG compared with the CG, including smooth expectoration (76.2% vs. 75.0%), decreased secretions (61.9% vs. 58.3%), reduced viscosity of secretions (66.7% vs. 58.3%), lower cough frequency (71.4% vs. 50.0%), improved sore throat (52.4% vs. 41.7%), and swallowing function (52.4% vs. 50.0%). The continuation of chemo-radiotherapy without disruption was higher in the RG than it was in the CG (66.7% vs. 50.0%). There was no significant difference in adverse effects between the two groups. *Conclusions*: Adjuvant respiratory therapy not only improves secretion expectoration, but also reduces side effects, thus promoting the completion of the CCRT schedule in patients with head and neck cancer.

## 1. Introduction

The treatment of head and neck cancer mainly depends on the tumor location, extent, severity, and type. Most tumors are treated by surgery combined with radiation therapy or chemotherapy. Most treatment methods for head and neck cancer have a considerable impact on the patient’s upper respiratory tract and digestive tract, including breathing, speaking, eating, and drinking. Active treatment of head and neck cancer may also cause damage to the normal cells of the oral cavity via both direct and indirect toxic effects [1,2]. Patients with mucositis often have reductions or breaks imposed on the cytotoxic therapy, which may lead to an increased risk of infection and hospitalization, compounding the cost of treatment as well as reducing survival rates [3].

Radiation therapy is one of the common treatment methods for head and neck tumors. Radiation therapy can damage the salivary glands, causing salivary cortex hypofunction and xerostomia, which often lead to mucositis, oropharyngeal dryness, dysphagia, swallowing pain, and changes in taste and smell, as well as other acute inflammatory reactions, which can lead to dehydration, dysgeusia, and malnutrition in patients. Studies have demonstrated that 38% of patients present mild-moderate to moderate-severe swallowing impairment [4]. Among which the incidence of dysphagia is about 30%, dry oropharyngeal and dysphagia can easily cause patients to suffer from aspiration or aspiration pneumonia, which is a life-threatening pulmonary complication [5,6,7,8,9]. Moreover, due to the limited function of the irradiated throat muscles and nerves in head and neck cancer patients, and the anatomical changes caused by the original tumor or surgery, the secretion or sputum accumulated in the throat cannot always be effectively removed [10,11].

Chest physical therapy is widely used for patients with airway diseases; the main goal of the intervention is to facilitate secretion transport, thereby decreasing secretion retention in the airways [12]. Previous studies showed that ß2 adrenergic agonist bronchodilators can improve mucociliary clearance via the alteration of airway ciliary motility, and anticholinergics can act on nerve pathways to reduce mucus secretion in patients with obstructive lung disease [13,14]. A recent study also showed that tiotropium decreases symptoms associated with sputum in chronic obstructive pulmonary disease (COPD) patients, an effect that may be related to the inhibition of airway mucus hypersecretion and an improvement in airway mucociliary clearance [15]. Moreover, tiotropium may alleviate the asthmatic cough refractory to inhaled corticosteroids and long-acting β2 agonists by modulating cough reflex sensitivity but not through bronchodilation [16]. Although chest respiratory therapy and adrenergic agonist/anticholinergics bronchodilators have been routinely used, alone or in combination, to enhance mucociliary clearance in patients with obstructive lung disease, the existing evidence does not consistently show their clinical effectiveness for patients with head and neck cancer. The aim of this study was to investigate the beneficial effects of auxiliary respiratory therapy, including bronchodilator inhalation plus respiratory control and sputum removal skills, for patients with head and neck cancer receiving CCRT.

## 2. Materials and Methods

We included patients with cancers of the oropharyngeal, laryngeal, hypopharyngeal, and oral cavity who were treated with CCRT from November 2016 to May 2018 in Chang Gung Memorial Hospital. The enrollment criteria for this study were: (1) aged between 20 and 70 years old, (2) diagnosed with a head and neck cancer, (3) prepared for aggressive CCRT, (4) with a good performance status, ECOG score: 0–1, and (5) cooperated with adjuvant respiratory therapy. The exclusion criteria were: (1) poor consciousness, (2) diagnosed with an infectious disease, such as open tuberculosis, (3) the patient or caregiver was not considered an adequate collaborator, and (4) patients with a tracheostomy. This retrospective study was approved by the Chang Gung Memorial Hospital’s institutional review board/ethics committee.

Patients were divided into the control group (CG) or the research group (RG) according to their respiratory therapy in the medical record. The course of adjuvant respiratory therapy was 5 times every week during the CCRT period.

In the CG, patients were treated with routine breathing exercises and taught sputum expelling techniques. The procedure was to first ask the patient to do pursed-lip breathing and to slowly inhale from the nose, then to adopt diaphragmatic breathing, protrude the abdomen when inhaling, slowly purse the lips, and exhale. Patients were taught to adjust their breathing pattern with the time ratio of inhalation to exhalation about 1:3 or 1:4, and to then breathe or directly cough, and to repeat for 3–4 cycles. The entire process took about 10–15 min to complete, once a day, 5 times a week, until the end of the radiation course.

Patients in the RG were treated with the inhalation of bronchodilator agents, in addition to the treatment administered in the CG. Before each radiotherapy, the patients went to the Respiratory Therapy Recovery Center to receive medical inhalation therapy, respiratory control, and sputum removal techniques. The patients would sit and inhale a total of 4 mL Terbutaline 5 mg (2 mL) and Ipratropium 0.5 mg (2 mL). After the drug was inhaled, breathing exercises and sputum removal techniques were taught as described above for the CG. The whole process took about 20–30 min to complete, once a day, 5 times a week, until the end of the radiation course.

All the above treatments were routine respiratory therapies in our hospital and were decided by a clinical physician. In the treatment process, the clinical physician accompanied the patient throughout the process, and any changes in the patient’s vital signs and responses during the treatment were monitored and recorded. The treatment was stopped immediately if the patient had any of the following conditions during the process: (1) heart rate increased more than 20% above baseline for 3 min, (2) systolic blood pressure (SBP) was >180 mmHg or <80 mmHg, (3) persistent bronchospasm despite inhalation of one bronchodilator (Terbutaline 5 mg), (4) active tumor bleeding, (5) infection symptoms, such as fever ≥38.5 °C (ear temperature), or (6) the patient requested to stop the treatment course.

Demographic covariates were obtained, including the patient’s age at diagnosis, gender, height, weight, and surgery status. Chemo-radiation therapy compliance after respiratory therapy was recorded.

A questionnaire was routinely used to evaluate the patient’s respiratory treatment benefit every week after treatment (from visit 1 to visit 3), including (1) question 2: the effect of inhaled medication on the secretion of expectoration (Q2.1), secretion viscosity (Q2.2), secretion amount (Q2.3), cough frequency (Q2.4), cough severity (Q2.5), sleep quality (Q2.6), sore throat (Q2.7), swallowing function (Q2.8), and radiotherapy process (Q2.9). (2) Question 3: side effects after inhaled medication, including dry mouth (Q3.1), palpitation (Q3.2), worse cough (Q3.3), and increased secretion (Q3.4). (3) Question 4: personal barriers for respiratory care, such as scheduling troubles (Q4.1), distance (Q4.2), and financial burden (Q4.3). (4) Question 5: the beneficial effect of breathing therapy (Q5.1). The questionnaire was a Likert scale questionnaire that used a five-point agreement scale to collect data. The psychometric response scale consisted of five agree/disagree points, which were as follows: (1) strongly disagree; (2) disagree; (3) neither agree nor disagree; (4) agree; and (5) strongly agree, to provide detailed data about the patient’s opinions and attitudes regarding the level of agreement for the treatment outcomes.

Clinical characteristics and treatment outcomes were compared between patients with and without inhalation of bronchodilator agents using the student’s *t* test. A 2-sided *p* value of <0.05 was considered to indicate a statistically significant difference. Statistical analyses were performed using GraphPad Prism version 8 (GraphPad Software, La Jolla, CA, USA) and IBM SPSS Statistics 26 (SPSS, Chicago, IL, USA).

## 3. Results

From November 2016 to May 2018, a total of 115 head and neck cancer patients were recruited, and 80 patients fulfilled the study criteria. There were 41 patients in the CG, and 39 patients in the research group.

**Figure 1:**
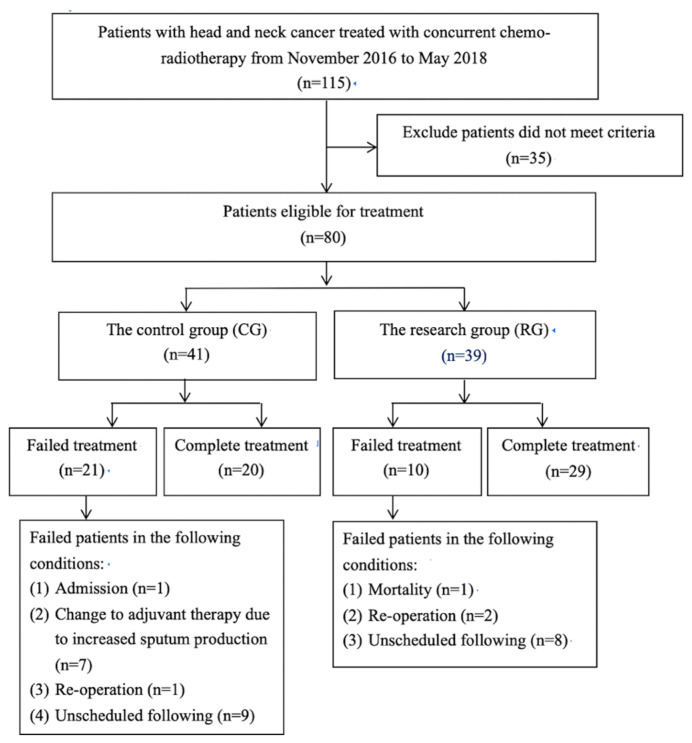
The total complete rate of treatment was significantly higher in the RG (29 patients) than it was in the CG (20 patients).

The total complete rate of treatment was significantly higher in the RG (29 patients) compared with the CG (20 patients; 74.4% vs. 48.8%, *p* < 0.001) (Figure 1). In the CG, seven patients were excluded because of increased sputum production and needed to receive adjuvant respiratory therapy. Table 1 shows the demographic characteristics of all patients who received complete treatment in both groups. Most patients in both groups were male. There was no significant difference in baseline characters between the two groups.

Based on the results of the questionnaire (Table 2), patients receiving inhaled therapy (RG) were aware of a significant reduction in the viscosity of their secretions by the second visit (Question 2.2, 3.79 ± 0.62 vs. 3.20 ± 0.77; *p* < 0.05). Inhaled therapy resulted in more dry mouth on the first and second visits (Question 3.1) and palpitation on the second visit (Question 3.2), compared with the CG; however, there was no significant difference in the rate of both side effects between the two groups on the third visit. Inhaled therapy also did not increase cough frequency and secretion (Question 3.3 and Question 3.4). Compared with the CG, significant personal barriers for respiratory care for the RG patients were scheduling troubles (Question 4.1) and financial burden (Question 4.3).

**Figure 2:**
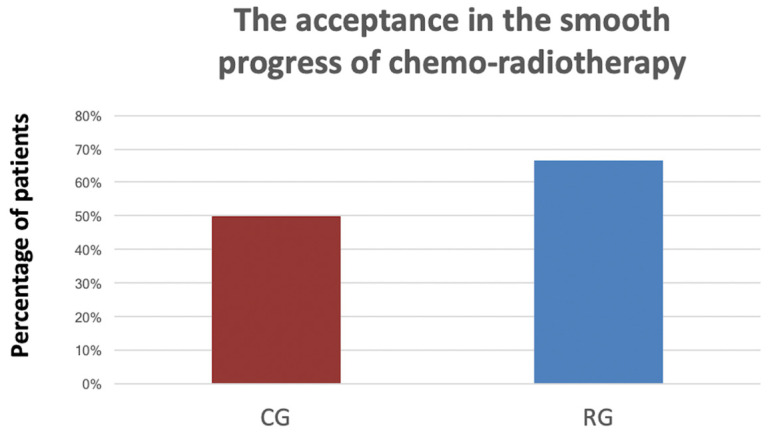
The acceptance in the smooth progress of chemo-radiotherapy was higher in the RG than it was in the CG.

Table 3 reveals the percentage of patients who agreed (score three) and strongly agreed (score five) for each question on the questionnaire, to evaluate the patients’ respiratory treatment benefit every week after treatment. On the third visit after therapy was completed, the effective rates of clinical symptoms were higher in the RG than in the CG, including smooth expectoration (76.2% vs. 75.0%), decreased secretions (61.9% vs. 58.3%), reduced viscosity of secretions (66.7% vs. 58.3%), lower cough frequency (71.4% vs. 50.0%), improved sore throat (52.4% vs. 41.7%), and swallowing function (52.4% vs. 50.0%) (Table 3). The acceptance in the smooth progress of chemo-radiotherapy was higher in the RG than it was in the CG (66.7% vs. 50.0%) (Question 2.9, Figure 2).

## 4. Discussion

Head and neck tumors are common malignant tumors, and the main treatment methods are surgery, radiation therapy, and chemotherapy. The morbidities associated with these therapies are very challenging for patients and their caregivers and can require life-long strategies to alleviate their deleterious effects on basic life functions and quality of life. However, there is a lack of evidence regarding adjuvant respiratory therapy intervention which aims to address the oral and pulmonary complications following head and neck cancer treatment. To the best of our knowledge, this is the first study to assess if implementing a combined inhalation medication and respiratory therapy program for the routine care of patients with head and neck cancer who are receiving CCRT would be feasible, adherent, and effective. We found that adjuvant respiratory therapy not only improves secretion expectoration but also reduces side effects, thereby promoting the completion of the CCRT schedule in patients with head and neck cancer.

The most common barriers of CCRT are the complications. Common oral complications of head and neck cancer after radiation therapy are mucositis, infections, saliva change, fibrosis, sensory dysfunctions, dental caries, periodontal disease, and osteoradionecrosis [17]. During and after cancer chemotherapy, patients are usually prone to dry mouth, dysphagia, difficulty opening their mouth, hoarse voice, sore throat, and oral mucosa damage after radiation exposure. Moreover, patients with head and neck cancer who are undergoing CCRT often experience pulmonary symptoms [9,18], resulting from aspiration, difficulty expelling sputum, and lung infection. Patients are often susceptible to a significant and abrupt deterioration in their therapy due to treatment-related side effects, thus leading to treatment incompletion and poor outcomes. Therefore, the major objective clinical result in this study is that our primary outcome demonstrated that adjuvant respiratory therapy increased treatment adherence to 72.4% in patients with advanced stage head and neck cancer receiving CCRT, who might now obtain a better outcome. However, this needs a long-term follow-up study to confirm.

Mucoactive agents have been the medication of choice for increasing patients’ ability to expectorate sputum and/or decrease mucus hypersecretion in the treatment of respiratory diseases where mucus hypersecretion is a clinical complication [19]. There is evidence supporting the use of β2-adrenergic agonists for enhancing mucociliary clearance [20,21]. β2-adrenergic agonists reduce the tone of bronchial smooth muscle and enhance the flow of mucus within lung airways [22]. Anticholinergic drugs are frequently used as mucoregulators. Anticholinergic medication, including atropine, ipratropium, scopolamine, glycopyrrolate, and tiotropium, block these secretory reflexes and reduce glandular output and sputum volume with the involvement of M1 and M3 receptors [23,24]. Although the combined inhalation of adrenergic agonist/anticholinergic bronchodilators has been used to enhance mucociliary clearance in patients with obstructive airway diseases, such as asthma and COPD [25,26,27], there are no previous reports concerning their clinical effectiveness on mucus secretion and the associated pulmonary symptoms for patients with head and neck cancer undergoing therapy. The results of the questionnaire presented the subjective results reported by patients in this study and showed that patients receiving inhaled therapy were aware of a significant reduction in the viscosity of their secretions and had a decrease in cough frequency and cough severity with a mild increase in dry mouth and palpitation, suggesting that adding inhaled bronchodilators to respiratory therapy is a feasible and effective treatment for advanced stage head and neck cancer patients receiving CCRT. Whether adjuvant respiratory therapy can reduce the incidence of aspiration and associated pneumonia needs further investigation.

The clinical safety of inhaled β2 agonists has been a source of controversy for decades [28]. β2 agonists have been associated with an increased risk of adverse cardiovascular events due to actions of the β1 receptor, which may cause tachycardia and palpitations [29]. The most common side effects of inhaled anticholinergics are dry mouth and, with aerosol administration using a poorly fitting mask, mydriasis; however, there are no adverse effects associated with mucus clearance or viscosity [30]. Data regarding their risk is conflicting, but caution is advised when using inhaled β2 agonists in patients with preexisting cardiovascular disease [31]. In this study, we found that after receiving inhaled therapy, patients became cognizant of a mild increase in dry mouth and palpitation, but there was not an increase in cough severity and secretion, which is consistent with the findings of previous studies. This suggests that adding inhaled bronchodilators to respiratory therapy is a safe strategy for advanced stage head and neck cancer patients receiving CCRT. In addition, our study indicated that the significant personal barriers associated with adjuvant respiratory therapy for head and neck cancer patients were not the side effects of treatment but scheduling troubles and financial burden. These primary problems should be addressed in future studies.

In this study the major objective clinical outcome was the adherence of CCRT, and the questionnaire was used to assess the subjective results. Although no profound effects of adjuvant respiratory therapy were seen on the various objective outcome parameters in this study, we demonstrated via our primary outcome that adjuvant respiratory therapy is feasible, safe, and well tolerated by patients with advanced stage head and neck cancer, and that it can enhance the treatment adherence and completion of CCRT. Secondary outcomes, as measured by a self-reported questionnaire, were achieved, including a significant reduction in the viscosity of the secretion and a decreasing trend in cough frequency and cough severity. The potential benefits of using adjuvant respiratory therapy in patients receiving CCRT on long-term outcomes and survival can only be evaluated in a larger sample and long-term study designs. In addition, it is also important to further evaluate these outcomes and whether adjuvant respiratory therapy could be used as a preventive support for long-term manifestations of head and neck cancer-related side effects.

Adjuvant respiratory therapy can significantly improve the compliance of patients receiving CCRT. However, some limitations and questions remain to be resolved: (1) whether respiratory therapy should be given early or only when there are problems, (2) how treatment compliance can be improved, (3) whether respiratory therapy improves long-term tumor treatment control and survival rates, and (4) whether improper use increases the risk of nosocomial infection. These are all topics that require further investigation. Moreover, as this is a retrospective study, the treatment protocol is dependent on shared decision-making with patients, for example, the self-selection and compliance of patients, as well as their socioeconomic status, thus it might lead to potential sources of bias. This study enrolled a relatively small number of subjects in each group, which may limit the validity of inferred results. Therefore, prospective enrollment and an increased number of subjects should be conducted in future studies.

Tumor location may play a critical role in respiratory symptom burden and the effectiveness of supportive interventions. Based on our clinical observations, patients with oral tongue cancer—particularly those who had undergone surgery—demonstrated greater difficulty in secretion clearance, likely due to anatomical alterations and impaired tongue mobility. These patients appeared to derive a greater benefit from adjuvant respiratory therapy. However, due to the limited sample size in our study, meaningful statistical stratification by tumor subsite could not be reliably performed. Future prospective studies with larger cohorts are warranted to validate these findings and clarify the influence of tumor location on treatment response.

Patients with a tracheotomy often experience more complex and severe respiratory complications. The exclusion of patients with a tracheotomy in our study may limit the generalizability of the study’s conclusions. Addressing this subgroup in future studies would provide a more comprehensive understanding of respiratory risks in this population, thus further enhancing the quality and impact of our study.

## 5. Conclusions

In conclusion, this is one of the first studies to investigate the role of adjuvant respiratory therapy in patients with head and neck cancer receiving CCRT. Adjuvant respiratory therapy was shown to not only improve secretion expectoration but to also reduce side effects, thereby promoting the completion of the treatment schedule of CCRT in patients with head and neck cancer. Our findings suggest that adjuvant respiratory therapy in patients undergoing CCRT for head and neck cancer was effective, safe, and well tolerated by patients, as indicated by a good adherence level. Further studies with a larger sample size are required to reveal additional potential effects of adjuvant respiratory therapy during CCRT in head and neck cancer patients.

## Figures and Tables

**Table 1:** Demographic characteristics of patients with complete treatment.

	CG (*n* = 20)	RG (*n* = 29)	*p* Value
Male:Female	20:0	26:3	0.04
Age	54.1 ± 7.9	52.2 ± 9.2	0.23
Body height (cm)	165 ± 5.3	166 ± 7.8	0.27
Body weight	65.5 ± 8.6	68.4 ± 12.3	0.17
BMI	24.1 ± 3.3	24.6 ± 3.7	0.32
Alcohol	15 (75%)	26 (89.7%)	0.37
Smoke	19 (95%)	24 (82.8%)	0.08
Betel nut	16 (80%)	17 (58.6%)	0.15
Surgery	10 (50%)	20 (69%)	0.10
Hypertension	4 (20%)	10 (34.5%)	0.13
DM	3 (15%)	6 (20.7%)	0.31
Family history	0	1 (3.4%)	0.16

CG: the control group; RG: the research group. Note: Values are displayed as mean (SD) or total (%). Abbreviations: BMI, Body Mass Index; DM, Diabetes Mellitus.

**Table 2:** Results of the questionnaire to evaluate the patient’s respiratory treatment benefit every week after treatment.

Question	Treatment	CG (*n* = 20)	RG (*n* = 29)	*p*-Value
Question 2.1: I feel that after the medicine is inhaled, it can help to expel secretion smoothly	1st	4.05 ± 0.83	4.00 ± 0.85	0.838
	2nd	3.70 ± 0.80	3.90 ± 0.56	0.316
	3rd	4.00 ± 0.79	4.03 ± 0.73	0.876
Question 2.2: I feel that after the medicine is inhaled, the viscosity of the secretion is reduced	1st	3.65 ± 0.81	3.69 ± 0.81	0.867
	2nd	3.20 ± 0.77	3.79 ± 0.62	0.004
	3rd	3.65 ± 0.81	3.75 ± 0.79	0.641
Question 2.3: I feel the amount of secretion decreases after the medicine is inhaled	1st	3.45 ± 0.94	3.83 ± 0.84	0.150
	2nd	3.55 ± 1.05	3.76 ± 0.64	0.391
	3rd	3.30 ± 0.86	3.62 ± 0.73	0.782
Question 2.4: I feel that after the medicine is inhaled, frequency of cough decreases	1st	3.55 ± 0.83	3.90 ± 0.86	0.165
	2nd	3.30 ± 0.86	3.76 ± 0.79	0.060
	3rd	3.60 ± 0.88	3.76 ± 0.79	0.512
Question 2.5: I feel that after inhaling the medicine, the severity of cough is reduced	1st	3.55 ± 0.89	3.83 ± 0.76	0.246
	2nd	3.32 ± 0.89	3.76 ± 0.79	0.076
	3rd	3.75 ± 0.79	3.59 ± 0.87	0.503
Question 2.6: I feel the quality of sleep improves after the medicine is inhaled	1st	3.40 ± 0.99	3.69 ± 0.89	0.291
	2nd	3.35 ± 0.99	3.55 ± 0.91	0.465
	3rd	3.65 ± 0.93	3.59 ± 0.82	0.802
Question 2.7: I feel that the sore throat improves after the medicine is inhaled	1st	3.40 ± 1.10	3.52 ± 0.95	0.692
	2nd	3.35 ± 0.88	3.66 ± 0.77	0.203
	3rd	3.55 ± 0.83	3.48 ± 1.00	0.804
Question 2.8: I feel the swallowing function improves after the medicine is inhaled	1st	3.45 ± 1.00	3.21 ± 1.01	0.411
	2nd	3.10 ± 0.85	3.38 ± 0.78	0.240
	3rd	3.55 ± 0.83	3.34 ± 1.00	0.456
Question 2.9: I feel the inhalation of the medicine will help the radiotherapy process go smoothly	1st	3.45 ± 1.00	3.75 ± 0.69	0.206
	2nd	3.30 ± 0.92	3.69 ± 0.60	0.080
	3rd	3.55 ± 0.94	3.83 ± 0.76	0.261
Question 3.1: I feel dry mouth will increase after inhalation	1st	2.90 ± 0.91	3.69 ± 0.90	0.004
	2nd	3.00 ± 0.86	3.66 ± 0.67	0.004
	3rd	2.85 ± 0.81	3.41 ± 0.82	0.022
Question 3.2: I feel the inhalation of the medicine will increase the palpitations	1st	2.50 ± 0.83	3.00 ± 1.03	0.079
	2nd	2.40 ± 0.68	3.69 ± 0.60	0.000
	3rd	2.45 ± 0.69	2.83 ± 0.85	0.105
Question 3.3: I feel that after inhaling the medicine, it makes the cough worse	1st	2.40 ± 0.94	2.17 ± 0.60	0.306
	2nd	2.30 ± 0.66	2.34 ± 0.61	0.808
	3rd	2.25 ± 0.64	2.34 ± 0.48	0.557
Question 3.4: I feel that after inhaling the medicine, it increases the amount of secretion	1st	2.55 ± 0.83	2.31 ± 0.85	0.331
	2nd	2.50 ± 0.83	2.48 ± 0.74	0.939
	3rd	2.30 ± 0.66	2.49 ± 0.63	0.333
Question 4.1: Receiving respiratory care will increase scheduling troubles	1st	2.60 ± 0.94	2.72 ± 0.96	0.656
	2nd	2.80 ± 0.95	3.83 ± 0.76	0.000
	3rd	2.45 ± 0.69	3.90 ± 0.56	0.000
Question 4.2: Receiving respiratory care, I feel the journey is far away	1st	2.70 ± 0.86	2.34 ± 0.61	0.099
	2nd	2.80 ± 0.95	2.52 ± 0.78	0.262
	3rd	2.55 ± 0.83	0.44 ± 0.69	0.641
Question 4.3: Receiving respiratory care will increase the financial burden	1st	2.25 ± 0.79	2.00 ± 0.65	0.232
	2nd	2.50 ± 0.76	2.03 ± 0.63	0.023
	3rd	2.35 ± 0.75	2.17 ± 0.47	0.310
Question5.1: After the whole course of treatment, I feel that breathing therapy is helpful to me	1st	3.85 ± 0.81	3.82 ± 0.76	0.922
	2nd	3.80 ± 0.83	4.00 ± 0.60	0.332
	3rd	4.00 ± 0.79	4.10 ± 0.67	0.626

**Table 3:** Percentage of patients with agree and strongly agree of each question based on the questionnaire to evaluate the patients’ respiratory treatment benefit every week after treatment.

Question	Treatment	CG (*n* = 20)	RG (*n* = 29)
Question 2.1: I feel that after the medicine is inhaled, it can help to expel secretion smoothly	1st	83.3%	71.4%
	2nd	58.3%	76.2%
	3rd	75.0%	76.2%
Question 2.2: I feel that after the medicine is inhaled, the viscosity of the secretion is reduced	1st	50.0%	57.1%
	2nd	41.7%	66.7%
	3rd	58.3%	66.7%
Question 2.3: I feel the amount of secretion decreases after the medicine is inhaled	1st	50.0%	61.9%
	2nd	41.7%	76.2%
	3rd	58.3%	61.9%
Question 2.4: I feel that after the medicine is inhaled, frequency of cough decreases	1st	50.0%	57.1%
	2nd	33.3%	66.7%
	3rd	50.0%	71.4%
Question 2.5: I feel that after inhaling the medicine, the severity of cough is reduced	1st	58.3%	66.7%
	2nd	33.3%	71.4%
	3rd	66.7%	61.9%
Question 2.6: I feel the quality of sleep improves after the medicine is inhaled	1st	50.0%	71.4%
	2nd	41.7%	61.9%
	3rd	41.7%	52.4%
Question 2.7: I feel that the sore throat improves after the medicine is inhaled	1st	66.7%	66.7%
	2nd	41.7%	61.9%
	3rd	41.7%	52.4%
Question 2.8: I feel the swallowing function improves after the medicine is inhaled	1st	50.0%	42.9%
	2nd	25.0%	57.1%
	3rd	50.0%	52.4%
Question 2.9: I feel the inhalation of the medicine will help the radiotherapy process go smoothly	1st	58.2%	66.7%
	2nd	33.3%	61.9%
	3rd	50.0%	66.7%
Question 3.1: I feel dry mouth will increase after inhalation	1st	41.7%	71.4%
	2nd	33.3%	57.1%
	3rd	33.3%	33.3%
Question 3.2: I feel the inhalation of the medicine will increase the palpitations	1st	16.7%	28.6%
	2nd	0.0%	14.3%
	3rd	16.7%	9.5%
Question 3.3: I feel that after inhaling the medicine, it makes the cough worse	1st	16.7%	9.5%
	2nd	0.0%	4.8%
	3rd	8.3%	0.0%
Question 3.4: I feel that after inhaling the medicine, it increases the amount of secretion	1st	16.7%	9.5%
	2nd	8.3%	9.5%
	3rd	8.3%	4.8%
Question 4.1: Receiving respiratory care will increase scheduling troubles	1st	25.0%	19.0%
	2nd	16.7%	9.5%
	3rd	8.3%	9.1%
Question 4.2: Receiving respiratory care, I feel the journey is far away	1st	25.0%	0.0%
	2nd	16.7%	9.5%
	3rd	16.7%	9.1%
Question 4.3: Receiving respiratory care will increase the financial burden	1st	8.3%	0.0%
	2nd	8.3%	0.0%
	3rd	8.3%	0.0%
Question 5.1: After the whole course of treatment, I feel that breathing therapy is helpful to me	1st	66.7%	71.4%
	2nd	75.0%	81.0%
	3rd	75.0%	77.3%

## Data Availability

The data sets analyzed during the current study are available from the corresponding author upon reasonable request.
